# Rearing in an envy-like environment increases anxiety-like behaviour in mice

**DOI:** 10.1515/tnsci-2022-0364

**Published:** 2025-02-22

**Authors:** Hiroshi Ueno, Eriko Kitano, Yu Takahashi, Sachiko Mori, Shinji Murakami, Kenta Wani, Yosuke Matsumoto, Motoi Okamoto, Takeshi Ishihara

**Affiliations:** Department of Medical Technology, Kawasaki University of Medical Welfare, 288, Matsushima, Kurashiki, Okayama, 701-0193, Japan; Department of Psychiatry, Kawasaki Medical School, Kurashiki, 701-0192, Japan; Department of Neuropsychiatry, Graduate School of Medicine, Dentistry and Pharmaceutical Sciences, Okayama University, Okayama, 700-8558, Japan; Department of Medical Technology, Graduate School of Health Sciences, Okayama University, Okayama, 700-8558, Japan

**Keywords:** behaviour, anxiety, mouse, envy, rodent

## Abstract

Interest in the societal and psychological harm caused by widespread envy and social comparison is increasing. Envy is associated with anxiety and depression, though the mechanism by which envy affects neuropsychiatric disorders, such as depression, remains unclear. Clarifying the neurobiological basis of envy’s effects on behaviour and emotion regulation in experimental mice is essential for developing disease-prevention and treatment strategies. As mice recognize other mice in neighbouring cages, this study investigated whether they recognize neighbouring cages housed in environmentally enriched cages and suffer psychological stress due to envy. After being raised in an envy-like environment for 3 weeks, we revealed changes in the behaviour of the mice through a series of behavioural experiments. Mice raised in an envious environment showed increased body weight and anxiety-like behaviour but decreased social behaviour and serum corticosterone levels compared to control mice. Thus, mice recognize their neighbouring cages and experience psychological stress due to envy. This study revealed a part of the scientific basis for why envy increased anxiety. Using this novel experimental breeding environment, it may be possible to create an experimental animal model of anxiety disorders.

## Abbreviations

ANOVA: analyses of variance
HPA: hypothalamic–pituitary–adrenal
PS: Porsolt forced swim test
TS: tail suspension test

## 1 Introduction

An estimated 4 billion people currently use at least one social media platform, and statistics show that people worldwide spend an average of more than 2 h per day on social media [1,2]. Moreover, the rise of social media has increased the interest in the societal psychological harm that can be caused through widespread envy and social comparison [3–6]. One hypothesis to better understand the harmful effects of social networking services assigns a prominent role to envy and social comparison [3,7]. Envy and social comparison are thought to be closely intertwined according to the theory that people are fundamentally motivated to evaluate themselves with reference to social information when objective parameters are unavailable [8–10]. Self-reported measures of envy have been reported to be negatively correlated with self-esteem and are positively correlated with the incidence of depression [11]. Similarly, Gold reported an association of envy with anxiety and depression [12].

Jealousy and envy are commonly used words with similar meanings; however, psychologically, they are two different emotions [13]. Envy is the feeling of anger that someone other than oneself owns and enjoys something desirable and good, and is based on a dyadic relationship. Jealousy, on the other hand, is an emotion in which one is afraid that the object of one's love will become attached to another being in a three-way relationship and is, therefore, jealous and hateful of that other being. Envy is a desire for superior qualities, achievements, or assets that one does not possess or a desire for the object to lose them [14]. Envy can be seen in various social situations, such as envy among work colleagues, siblings, and friends. Feelings of envy in this situation occur in the form of feelings of inferiority, defeat, humiliation, helplessness, mental pain, lack of self-esteem, decreased self-esteem, and a sense of well-being when comparing oneself to the object of envy.

Envy has been described as a universal motive related to social comparison [8]. Envy is a social emotion that focuses on problematic comparisons with others and the perceived poor performance of the self as compared with certain individuals. Social comparison theory states that people generally choose to compare themselves with others who have similar abilities and opinions [8]. Although people wish to avoid comparisons, they still routinely evaluate their position in comparison with relevant colleagues and even those closest to them [15,16]. People generally objectively perceive themselves as better than they seem to be [17]. When people feel inferior to someone else, they feel shame about their inadequacies. Most people's self-esteem is derived by comparing themselves with others.

Furthermore, envy is a stressor in the workplace and is particularly associated with negative outcomes, such as turnover intentions, job dissatisfaction, and decreased organisational commitment [18]. Outside of the workplace, malicious envy is strongly associated with poor mental health [19]. The increase in the experience of envy at the societal level coincides with a stepwise increase in psychopathological symptoms [20]. Envy is associated with depression, anger, anxiety, rumination, and interpersonal hostility [3]. In recent years, clinical researchers have highlighted the need to understand the extent and nature of the relationship between envy and mental health; in particular, there is an urgent need to develop interventions designed to reduce envy and its effects [21]. However, the mechanism whereby envy affects neuropsychiatric disorders, such as depression, remains unclear.

Laboratory mice play a central role in animal models of human behavioural disorders [22]. Therefore, it is important to clarify the neurobiological basis of the effects of envy on the regulation of behaviour and emotion in experimental mice. The establishment of animal models of neuropsychiatric disorders caused by chronic envy is essential to develop prevention and treatment strategies for these disorders. Humans and other animals have a sense of fairness. In particular, the idea that people react unfavourably to the unequal distribution of rewards is called inequity aversion and has been frequently investigated [23]. Recent reports indicate that primates, dogs, crows, and rodents (e.g. rats and mice) respond negatively to unequal conditions [24–27]. In the first step towards the aversion of inequality, an organism must be able to recognize the reward of others and compare it with the magnitude of its reward [28]. Accordingly, organisms that exhibit aversion to unfairness recognize the environment of others and compare it with their own. Considering these findings, mice possibly make social comparisons with others and experience envy.

In the present study, we investigated whether mice in regular cages recognize and envy others in environmentally enriched cages based on social comparisons with themselves. Mice recognize mice in neighbouring cages [29]. Therefore, it is possible that the environment wherein they are kept may have some effect on the mice.

This study aimed to clarify the effects of environmental enrichment on the behaviour of test mice when they recognize mice in neighbouring cages and whether an environment that causes envy is stressful for mice. Through this research, we hope to not only help establish a mouse model for envy-induced neuropsychiatric disorders but also facilitate the development of therapeutic drugs for anxiety disorders and depression that are caused by different developmental factors.

## 2 Methods

### 2.1 Animals

Nine-week-old C57BL/6N male mice were purchased from CLEA Japan (Tokyo, Japan) and housed in cages (five animals per cage). Transparent plastic cages (220 mm × 340 mm × 150 mm) with wire tops were used, and a nonwoven filter cap was attached to the top of the wire. The cages included the provision of nesting material with food (MF-R; ORIENTAL YEAST, Tokyo, Japan) and water *ad libitum*, under 12-h light–dark conditions (lights on at 8:00, lights off at 20:00), with a temperature maintained between 23 and 26°C. As behavioural variability is partially sex-dependent and because this study did not aim to compare the behaviours of males and females, only male mice were included.

### 2.2 Rearing in an envy-like environment

**Figure 1:**
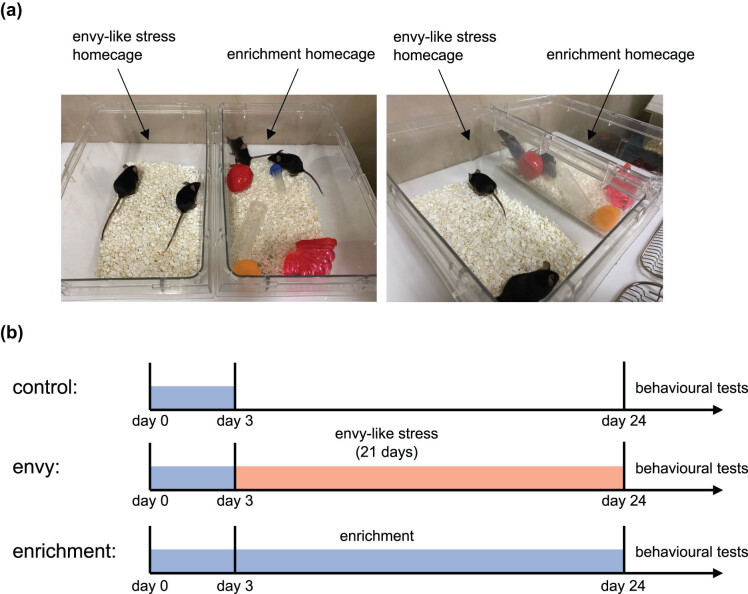
Experimental procedure. Envy-like environment-rearing situation with adjacent cages. (a) Subject mice were housed next to mice housed in environmental enrichment devices. This cage position was maintained for 3 weeks. (b) Time schedule showing all steps of the experimental treatment. Presentation of enrichment devices was carried out from day 0 to day 3. After 3 weeks of rearing in an envy-like environment, behavioral experiments were conducted.

Male C57BL/6N mice were randomly divided into three groups (control, envy-like stress, and enrichment) and placed in adjacent cages (https://www.randomizer.org). First, the enrichment equipment (two tubes for mice to put in, three objects) was placed in the cages of all groups for 3 days to allow the mice to familiarize themselves with the enrichment equipment (Figure 1a and b), which was left in the cages of mice in the enrichment group. To prevent boredom, the three objects in the enrichment group cages were replaced with new ones every 3 days. Mice in the envy-like stress group were housed in cages adjacent to those in the enrichment group, whereas mice in the control group were housed in opaque cages so that the surroundings of the cages could not be observed. To chronically stress the mice, we maintained them in this environment for 3 weeks. After 3 weeks, the mice underwent behavioural testing (Figure 1b).

### 2.3 Behavioural tests

All behavioural tests were conducted in behavioural testing rooms between 09:00 and 16:00, during the light phase of the light/dark cycle. After the tests, the equipment and toys were cleaned with 70% ethanol and superhypochlorous water to avoid artefacts caused by lingering olfactory cues. Behavioural tests were performed on naïve mice in accordance with the experimental order described further.

### 2.4 Grip-strength test

Neuromuscular strength was examined using the grip-strength test. Forelimb muscle strength was measured by using a grip dynamometer. Each mouse was lifted by its tail such that its front paws could grip the wire grid of the dynamometer. Subsequently, the mouse was slowly pulled back until the grid was released. The peak force (Newton, cN) exerted by the forelimbs was recorded.

### 2.5 Hotplate test

A hotplate test was used to assess nociception. Mice were placed on a plate heated to 55.0 ± 0.3°C, and the latency to the first paw response was recorded. Valid responses included shaking and paw licking. A latency period of 30 s was defined as complete analgesia and was used as the cut-off time to prevent tissue damage.

### 2.6 Light–dark transition test

The apparatus consisted of an acrylic cage (cm) divided into two sections of equal size using a partition with a door. One chamber had white acrylic walls and was brightly illuminated (200 lx) by lights above the ceiling, whereas the other chamber had black acrylic walls and was dark (50 lx). Both chambers had white plastic floors. Mice were placed in a dark chamber and allowed to move freely between the two chambers for 6 min with the door open. The distance travelled (*m*), total number of transitions, and time spent in the light chamber (s) were analysed using the ANY-MAZE software (ANY-MAZE, Stoelting Co., Wood Dale, IL, USA).

### 2.7 Y-maze test

Spatial working memory was measured using a Y-maze apparatus (arm length, 40 cm; bottom arm width, 3 cm; upper arm width, 10 cm; wall height, 12 cm). Each mouse was placed in the centre of the Y-maze for 6 min. Visual cues were placed around the maze in the testing chamber and remained there throughout the test period. The mice were tested without any previous exposure or habituation to the maze. The total distance travelled (*m*), the number of entries, and the number of turnovers were recorded and analysed using ANY-MAZE software.

### 2.8 Social interaction test in a novel environment

In the social interaction test, two mice from identical groups that were previously housed in different cages were placed together in a box (45 cm × 45 cm × 40 cm) and allowed to explore freely for 10 min [30]. Their behaviours were recorded. The total distance travelled, total number of contacts, and mean duration per contact were analysed using SMART version 3.0 (PanLab, Harvard Apparatus, Spain).

### 2.9 Sociability tests for novel objects and stranger mice using social interaction test apparatus

The apparatus was rectangular (45 cm  ×  45 cm  ×  40 cm). Two transparent cages (7.5 cm  ×  7.5 cm  ×  10 cm, with several 1 cm diameter holes) were placed at both ends of the rectangular apparatus (Figure 4d). Each mouse was placed in a box for 6 min and allowed to freely explore the habituation. In this test, a novel object was placed inside the cage, and an unfamiliar C57BL/6N male (stranger), who had no prior contact with the mice, was placed in another cage. The amount of time spent around each cage during the 6-min sessions was measured. The apparatus was then cleaned after each test phase. Data were recorded on video and analysed using ANY-MAZE software.

### 2.10 Tail-suspension test

Depressive-like behaviour was examined using the tail-suspension test. Each mouse was suspended by the tail in a white plastic chamber 60 cm above the floor and secured with adhesive tape placed <1 cm away from the tail tip. The resulting behaviour was recorded for 8 min using a video camera, and immobility time was measured. In this test, the immobility time was defined as the interval when the mice stopped struggling for ≥1 s. Data acquisition and analysis were performed using the ANY-MAZE software.

### 2.11 Porsolt forced-swim test

The Porsolt forced-swim test was used to assess depressive behaviour [31]. The apparatus comprised four Plexiglas cylinders (20 cm in height × 10 cm in diameter), which were filled with water (23°C) to a depth of 7.5 cm, based on previous studies. The mice were positioned in the cylinders for 8 min, and their behaviour was recorded. Consistent with the tail-suspension test, the immobility time was evaluated using ANY-MAZE software.

### 2.12 Corticosterone measurements

The mice were anaesthetized with a lethal dose of sodium pentobarbital (120 mg/kg, intraperitoneally). The mice were immediately sacrificed by decapitation under isoflurane anaesthesia, and truncal blood was collected in tubes. Blood was centrifuged at 0.8 × g for 10 min, and serum was collected and frozen at −80°C until analysis. Serum corticosterone concentrations were measured by using an enzyme-linked immunoassay (Cat. # K014-H5, Ann Arbor, Michigan, USA) according to the manufacturer’s instructions.

### 2.13 Statistical analysis

Statistical analyses were performed using GraphPad Prism (GraphPad Software Inc., San Diego, CA, USA). Normal distribution for all samples was assessed using the Shapiro–Wilk normality test prior to group analysis. Data were analysed using one-way analysis of variance (ANOVA), followed by Tukey’s test or two-way repeated-measures ANOVA, followed by Fisher’s least significant difference test or Student’s *t*-test. The data are presented as box plots. Statistical significance was defined as *p < 0.05 and ^+^ *p* < 0.1.


**Ethical approval:** The research related to animals’ use has complied with all the relevant national regulations and institutional policies for the care and use of animals. All animal experiments were performed in accordance with the ARRIVE guidelines (https://www.nc3rs.org.uk/arrive-guidelines) and the U.S. National Institutes of Health (NIH) Guide for the Care and Use of Laboratory Animals (NIH Publication No. 80-23, revised in 1996). This study was approved by the Committee for Animal Experiments at Kawasaki Medical School Advanced Research Centre. All efforts were made to minimize the number of animals used and their suffering. The use of animals was reduced via the experimental design, allowing statistically significant changes to be demonstrated, with the smallest number of animals per group and the smallest number of groups that were feasible for scientific rigor.

## 3 Results

### 3.1 Effects of envy-like stress on weight

**Figure 2:**
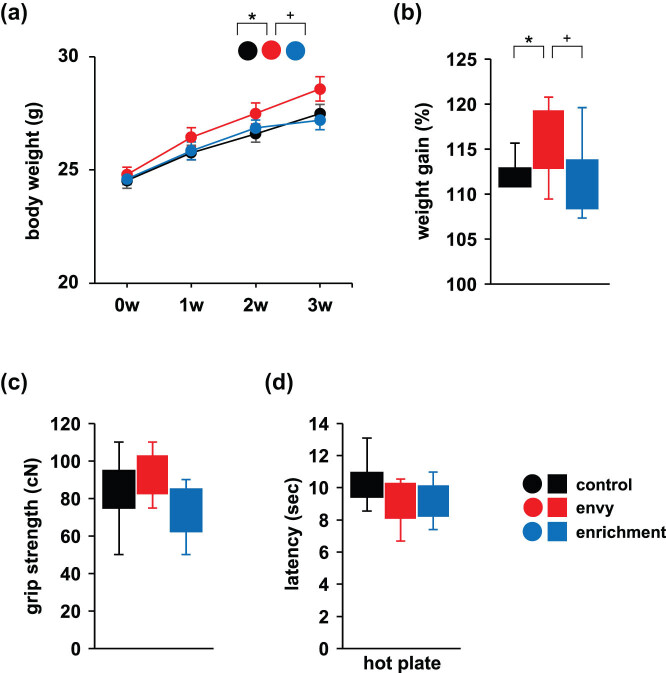
Effects of envy-like stress on physical characteristics of mice. (a) Mouse body weight measured weekly for 3 weeks. (b) Percentage change in mouse body weight after 3 weeks. (c) Grip strength test. (d) Hot plate test results. Data are presented as mean ± standard error (a) or box plots (b–d). control: *n* = 10, envy (envy-like stress caused by environmental enrichment presentation in adjacent cages): *n* = 11, enrichment (environmental enrichment cages): *n* = 10. *p < 0.05, ^+^ *p* < 0.1. The *p*-values were calculated using two-way repeated-measures ANOVA in (a) and ANOVA in (b–d). SEM: standard error of the mean.

Body weights of the mice were recorded weekly during the envy-like stress period. Compared to the control and enriched mice, the body weight of mice that were exposed to envy-like stress increased (Figure 2a; control vs envy: *p* = 0.020*; envy vs enrichment: *p* = 0.042*; control vs enrichment: *p* = 0.792, two-way repeated-measures ANOVA: group × time: *F*_4,8_ = 15.74, *p* = 0.008*). Compared with control mice, weight gain was higher in mice subjected to envy stress (Figure 2b; control vs envy: *p* = 0.048*; envy vs enrichment: *p* = 0.081^+^; control vs enrichment: *p* = 0.967, *F*_2,28_ = 0.0, *p* = 0.034*, ANOVA).

### 3.2 Effects of envy-like stress on grip strength and hotplate tests

The comparison of neuromuscular strength (grip-strength test) among the envy-like stress, enrichment, and control groups showed no significant intergroup difference (Figure 2c; control vs envy: *p* = 0.842; envy vs enrichment: *p* = 0.111; control vs enrichment: *p* = 0.327, *F*_2,28_ = 2.279, *p* = 0.121, ANOVA). Though mice from the envy-like stress, enrichment, and control groups were placed on a hotplate to assess nociception and chronic suppression of aggressive behaviours by heat pain, no significant intergroup difference in pain thresholds was observed (Figure 2d; control vs envy: *p* = 0.901; envy vs enrichment: *p* = 0.729; control vs enrichment: *p* = 0.481, *F*_2,28_ = 0.700, *p* = 0.505, ANOVA).

### 3.3 Effects of envy-like stress on anxiety-like behaviour in the light–dark transition test

**Figure 3:**
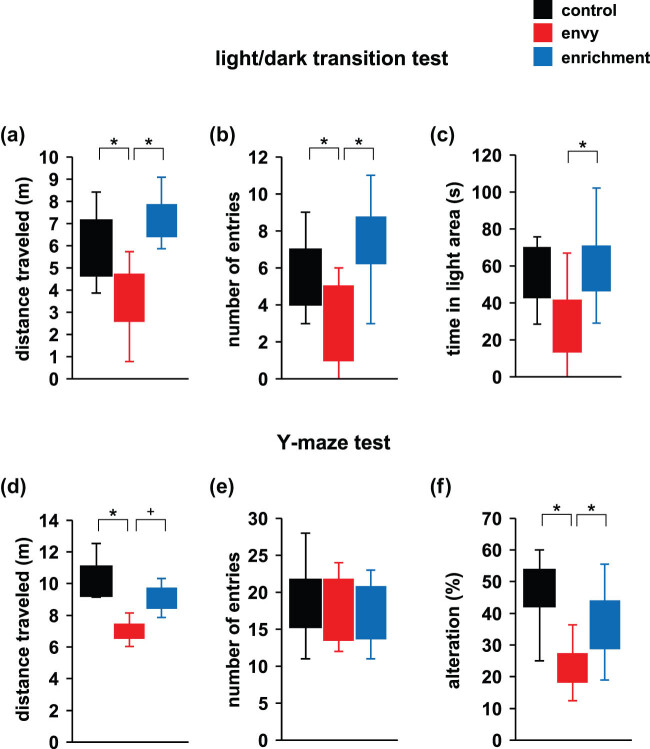
Effects of envy-like stress on anxiety-like behaviour and cognitive function. Total distance traveled (a), the number of entries into the light area (b), and time spent in the light area (c) in the light/dark transition test. Total distance travelled (d), total number of arm entries (e), and percentage of alternations (f). Data are presented as box plots (a–f). control: *n* = 10, envy (envy-like stress caused by environmental enrichment presentation in adjacent cages): *n* = 11, enrichment (environmental enriched cages), *n* = 10. *p < 0.05, ^+^ *p* < 0.1. *p*-values were calculated using ANOVA in (a–f).

In the envy-like stress group, anxiety-like behaviour was assessed using the light–dark transition test. Compared with the control group and enrichment group, the envy-like stress group travelled a shorter distance (Figure 3a; control vs envy: *p* = 0.018*; envy vs enrichment: *p* < 0.001*; control vs enrichment: *p* = 0.282, *F*_2,28_ = 10.97, *p* < 0.001*, ANOVA). Compared with the control and enrichment groups, the envy-like stress group entered the light area fewer times (Figure 3b; control vs envy: *p* = 0.027*; envy vs enrichment: *p* < 0.001*; control vs enrichment: *p* = 0.236, *F*_2,28_ = 10.34, *p <* 0.001*, ANOVA). Compared with the enrichment group, the envy-like stress group spent less time in the lit area (Figure 3c; control vs envy: *p* = 0.227; envy vs enrichment: *p* = 0.047*; control vs enrichment: *p* = 0.717, *F*_2,28_ = 3.289, *p* = 0.054*, ANOVA).

### 3.4 Effects of envy-like stress on cognitive function in the Y-maze test

In the envy-like stress group, we used the Y-maze test to assess short-term spatial working memory by monitoring spontaneous alternation behaviour. Compared with the control and enrichment groups, the distance travelled by the envy-like stress group was shorter (control vs envy: *p* = 0.009*; envy vs enrichment: *p* = 0.052^+^; control vs enrichment: *p* = 0.715, *F*_2,28_ = 5.623, *p* = 0.009*, ANOVA) or tended to decrease, respectively (Figure 3d). No significant intergroup differences were found for the number of arm entries (Figure 3e; control vs envy: *p* = 0.506; envy vs enrichment: *p* = 0.992; control vs enrichment: *p* = 0.579, *F*_2,28_ = 0.766, *p* = 0.475, ANOVA), though the envy-like stress group had a reduced proportion of alternations in the total entries as compared to the control and enrichment groups (Figure 3f; control vs envy: *p* = 0.004*; envy vs enrichment: *p* = 0.032*; control vs enrichment: *p* = 0.585, *F*_2,28_ = 7.098, *p* = 0.003*, ANOVA).

### 3.5 Effect of envy-like stress on social behaviour

**Figure 4:**
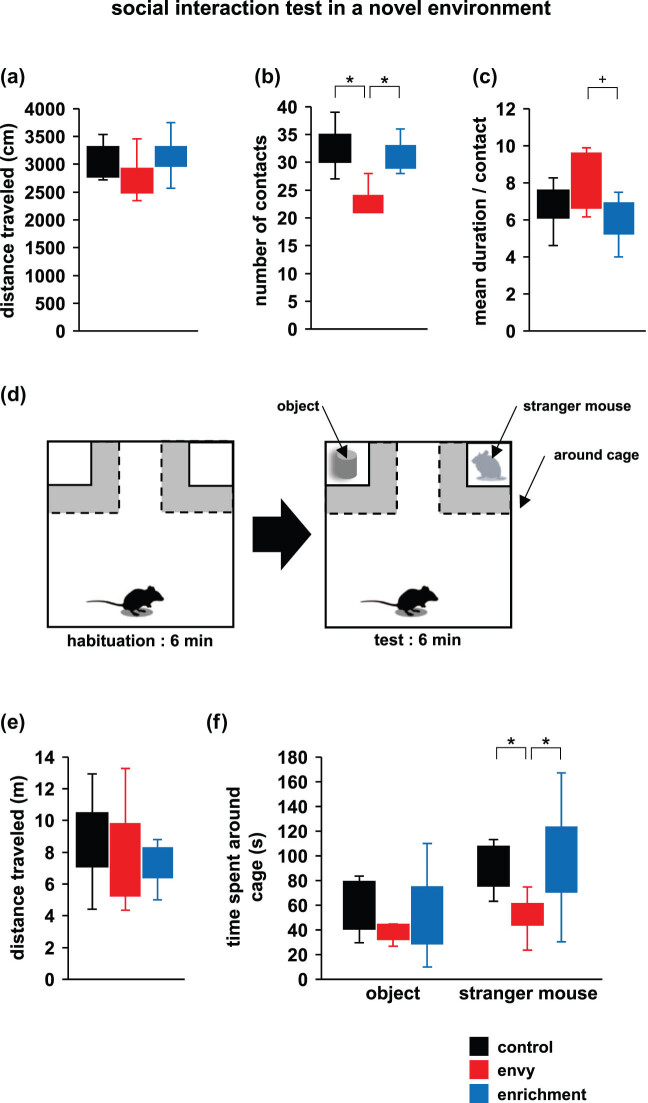
Effects of envy-like stress on social behaviour. Social interaction test in a novel environment: total distance traveled (a), total number of contacts (b), and mean duration per contact (c). Sociability tests for novel objects and stranger mice using social interaction test apparatus: Schematic diagram of the experiment (d), total distance traveled (e), and the amount of time spent around each cage (f). Data are presented as box plots (a–c, e, f). control: *n* = 10, envy (envy-like stress caused by environmental enrichment presentation in adjacent cages): *n* = 11, enrichment (environmental enrichment cages): *n* = 10. *p < 0.05, ^+^ *p* < 0.1. *p*-values were calculated using ANOVA in (a–c, e, f).

We used a social interaction test in a novel environment to investigate sociability in an envy-like stress group. There was no significant intergroup difference in the total distance travelled (Figure 4a; control vs envy: *p* = 0.392; envy vs enrichment: *p* = 0.337; control vs enrichment: *p* = 0.994, *F*_2,15_ = 1.375, *p* = 0.287, ANOVA). Compared to the control and enrichment groups, the envy-like stress group had fewer contacts (Figure 4b; control vs envy: *p* = 0.004*; envy vs enrichment: *p* = 0.012*; control vs enrichment: *p* = 0.773, *F*_2,15_ = 9.895, *p* = 0.003*, ANOVA), and there was no significant intergroup difference in the mean duration per contact (Figure 4c; control vs envy: *p* = 0.279; envy vs enrichment: *p* = 0.069^+^; control vs enrichment: *p* = 0.696, *F*_2,15_ = 3.185, *p* = 0.075*, ANOVA).

We investigated the intergroup difference in the interest in stranger mice and unfamiliar objects, which were placed in the cages at both ends of the experimental apparatus (Figure 4d), and found no significant intergroup difference in the total distance travelled (Figure 4e; control vs envy: *p* = 0.402; envy vs enrichment: *p* = 0.995; control vs enrichment: *p* = 0.454, *F*_2,28_ = 1.067, *p* = 0.358, ANOVA) and the duration spent around the strange-object cage (Figure 4f; object: control vs envy: *p* = 0.614; envy vs enrichment: *p* = 0.957; control vs enrichment: *p* = 0.784, *F*_2,28_ = 0.476, *p* = 0.626, ANOVA). Compared with the control and enrichment groups, the envy-like group spent significantly less time around the stranger mouse cage (Figure 4f; stranger: control vs envy: *p* = 0.048*; envy vs enrichment: *p* = 0.031*; control vs enrichment: *p* = 0.998, *F*_2,28_ = 4.598, *p* = 0.020*, ANOVA).

### 3.6 Effect of envy-like stress on depressive behaviour in the tail-suspension and Porsolt forced-swim tests

**Figure 5:**
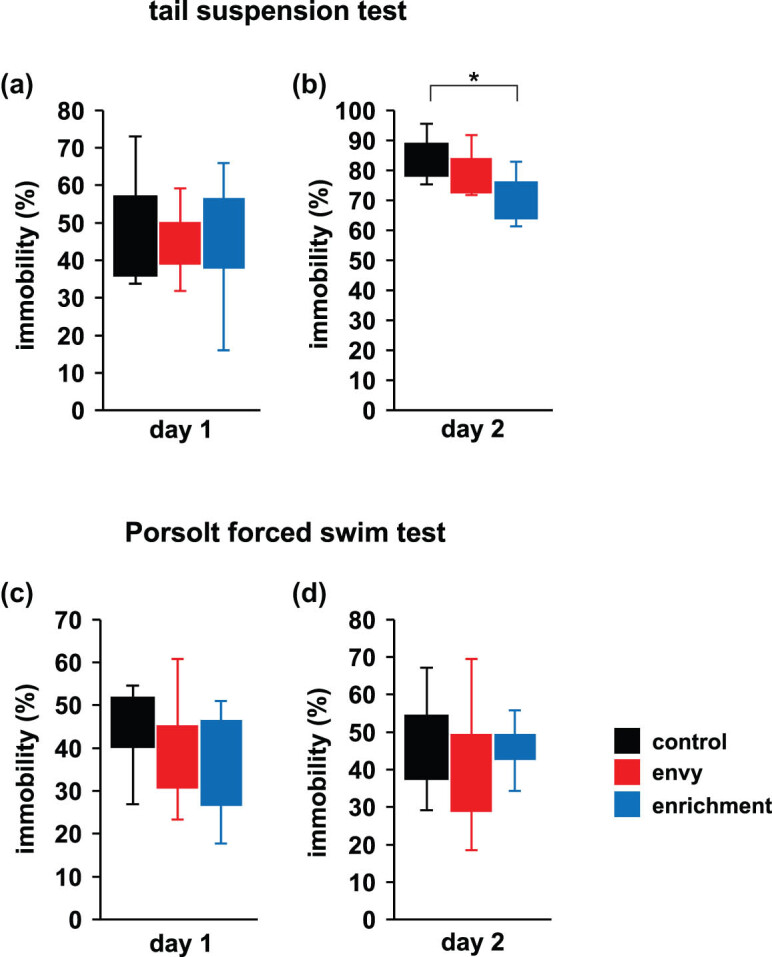
Effects of envy-like stress on depressive-like behaviour. Tail suspension test: the proportion of total time spent immobile on day 1 (a) and the proportion of total time spent immobile on day 2 (b). Porsolt forced swim test: the proportion of total time spent immobile on day 1 (c) and the proportion of total time spent immobile on day 2 (d). Data are presented as box plots (a–d). control: *n* = 10, envy (envy-like stress caused by environmental enrichment presentation in adjacent cages): *n* = 11, enrichment (environmental enrichment cages): *n* = 10. *p < 0.05, ^+^ *p* < 0.1. The *p*-values were calculated using ANOVA in (a–d).

Depressive behaviours were assessed using the Porsolt forced-swim and tail-suspension tests, and showed no significant intergroup difference in the immobility time in the tail-suspension test on Day 1 (Figure 5a; control vs envy: *p* = 0.939; envy vs enrichment: *p* = 0.989; control vs enrichment: *p* = 0.882, *F*_2,28_ = 0.121, *p* = 0.887, ANOVA) and Day 2 (Figure 5b; control vs envy: *p* = 0.291; envy vs enrichment: *p* = 0.355; control vs enrichment: *p* = 0.02*, *F*_2,28_ = 4.286, *p* = 0.026*, ANOVA); however, compared to that in the control group, the immobility time was significantly reduced in the enrichment group. There was no significant difference in the immobility time in the Porsolt forced-swim test (Figure 5c; control vs envy: *p* = 0.588; envy vs enrichment: *p* = 0.929; control vs enrichment: *p* = 0.377, *F*_2,28_ = 0.987, *p* = 0.386, ANOVA, Figure 5d; control vs envy: *p* = 0.628; envy vs enrichment: *p* = 0.779; control vs enrichment: *p* = 0.967, *F*_2,28_ = 0.4658, *p* = 0.632, ANOVA).

### 3.7 Effect of envy-like stress on serum corticosterone

**Figure 6:**
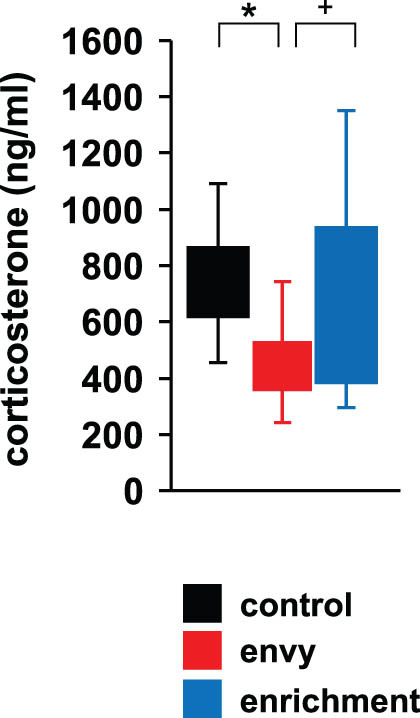
Effects of envy-like stress on serum corticosterone. Comparison of serum corticosterone levels. Data are presented as box plots. Control: *n* = 10, envy (envy-like stress caused by environmental enrichment presentation in adjacent cages): *n* = 11, enrichment (environmental enrichment cages): *n* = 10. *p < 0.05, ^+^ *p* < 0.1. *p*-values were calculated using ANOVA.

Serum corticosterone is a glucocorticoid that has a major role in regulating the stress response and increases with stress [32]. Compared with the control and enrichment groups, serum corticosterone levels were significantly and non-significantly lower, respectively, in the envy-like stress group (Figure 6; control vs envy, *p* = 0.029*; envy vs enrichment, *p* = 0.059^+^; control vs enrichment, *p* = 0.922, *F*_2,28_ = 4.486, *p* = 0.021*, ANOVA).

## 4 Discussion

This study of the behavioural changes of mice after exposure to an enviable breeding environment for 3 weeks showed that, compared to control mice, the mice that were raised in an enviable environment gradually gained weight, showed increased anxiety-like behaviour in the light–dark transition test, spent significantly less time interacting with others, and had lower serum corticosterone levels. These results indicated that an enviable environment alters mouse behaviour and serum corticosterone levels.

Mice that were housed in an envy-like environment gained weight as compared to other groups. Considerable changes in eating patterns and weight are common among people with depression, anxiety, or stress [33,34]. In humans, extremes in weight change, such as anorexia and weight loss, can occur in response to stress and depression because significant increases in food intake and body weight are very common [35,36]. In adolescents and young adults, mood disorders, anxiety, and weight gain are closely related and are recognized as common symptoms [37,38]. In animal models, stress is a well-known modulator of food intake and body weight. The social defeat model has been utilized to ascertain the aetiology of depression and anxiety, and weight gain during social defeat stress has been previously reported [39,40]. The results of the present study suggest that an enviable rearing environment may be stressful for mice, and weight gain secondary to increased food intake may occur.

The grip-strength test is ideal for measuring forelimb grip strength in mice [41]. However, the lack of a significant intergroup difference in grip strength suggests that the envious rearing environment has no effect on the muscle strength of mice. Using the hotplate test, a classic assay that reflects different modalities of thermal nociception [42], we measured analgesia in mice and found no significant intergroup difference. Thus, an envious rearing environment has no effect on pain sensitivity.

The light–dark transition test is a behavioural experiment that measures anxiety [43]. Mice that were exposed to an envy-like environment showed increased anxiety-like behaviour in the light–dark transition test, and therefore, an envy-like rearing environment possibly influences anxiety-like behaviour in mice. However, open-field, elevated-plus-maze, and light–dark box results may not measure the same type of anxiety-like behaviour [44]. For example, Balb/c mice behaved more “anxiously” in the elevated plus maze than in the open-field test [45,46]. Therefore, the lack of agreement between these two tests is not unusual [47].

The envious rearing environment decreased the distance travelled by mice in the Y-maze test and cognitive function. Besides the clear associations among memory, anxiety disorders, and impairment of skilled movements, an additional association may exist under physiological conditions. For example, when a healthy person is under pressure to perform an important task or activity, anxiety-like traits can affect athletic performance [48,49]. For example, anxiety may cause a pianist to make inaccurate movements [50]. Increased anxiety levels may, through predictive and/or attentional mechanisms, induce dual effects on skilled walking performance, alter movement control, and impair smooth movement [51]. Anxiety-like behaviour affects spatial memory tests in mice [52], and increased anxiety due to the envious rearing environment possibly reduced performance in the Y-maze test. To examine the extent to which cognitive function has actually improved, other behavioural experiments, such as the T-maze test [53], are needed.

In the social interaction test in a novel environment (one-chamber social interaction test), mice were exposed to an unfamiliar mouse in a chamber, and both mice were allowed to move freely [30]; herein, the number of contacts was significantly reduced as compared to that of mice raised in the envious environment and of control mice. Thus, the envy-like rearing environment reduced the social behaviour of mice.

The envy-like rearing environment did not change the mice's interest in novel objects, though it reduced their sociability with unfamiliar objects. This decrease in the approach time to unfamiliar mice may be attributable to an increase in anxiety-like behaviour. Compared to individuals without social anxiety, patients with social anxiety disorder experience higher levels of envy and exhibit lower sociability [54]. Social anxiety disorders, such as post-traumatic stress disorder, anxiety, and depression, are biological phenomena that are rooted in environmental influences. People with social anxiety exhibit a different pattern, with lower glucocorticoid levels and decreased reactivity later in life [55]. Mice raised in an envy-like environment exhibited behaviour similar to those reported previously on envy in humans, and this study suggests that mice may be affected by psychological effects similar to envy.

The tail-suspension test and forced-swim test are widely used to measure depression-like behaviour. Repeated exposure to social defeat stress induces a severe depression-like phenotype characterized by anhedonia, anxiety, and social avoidance behaviour [56,57]. Furthermore, chronic restraint stress increases depression-like behaviour [58]. However, the envy-like environment did not alter depression-like behaviour in mice, despite a significant positive correlation between envy and depression [59,60]. Envy can be defined as a complex emotion that involves a mixture of unpleasant, painful emotions, such as feelings of inferiority, hostility, and resentment [61,62]. Envy emerges as a contrastive response to unpleasant social comparisons [63], wherein the target is perceived as advantageous or superior, and the comparator feels unable to close the gap between himself and the target. Unlike previously reported stress in mice, it is unclear why the envy-like environment did not change depression-like behaviour. This may be because the existing stress is physical, whereas the envy-like environment confers psychological stress on mice. This study shows that envious rearing is not a stressor that increases depression-like behaviour in mice.

The hypothalamic–pituitary–adrenocortical (HPA) axis is an important component of the biological stress response that facilitates accommodation and adaptation to threats and traumatic exposures [64]. Exposure to stress triggers a series of HPA-activity events, which result in the release of the glucocorticoid hormone cortisol. Mice raised in an envy-like environment had decreased serum corticosterone levels. In rats, chronic predator odour stress-induced PTSD is accompanied by adrenal damage in the glucocorticoid-producing zona fasciculata and a decrease in plasma corticosterone levels [65]. Compared to those without PTSD, individuals with PTSD tend to have lower glucocorticoid levels [66]. In clinical and experimental studies, decreased glucocorticoid secretion and/or action accompanied adrenal insufficiency and, in several diseases, such as atypical depression, fibromyalgia, and chronic fatigue syndrome, neuroendocrine changes have been associated with impaired glucocorticoid signalling [67]. In individuals with chronic stress, plasma glucocorticoid levels decreased [68,69]. Dysregulation of the HPA-axis response to stress is a key feature of anxiety disorders [70]. Recent reports indicate that elevated cortisol confers a protective effect against anxiety levels; thus, cortisol or cortisol agonists have been suggested for the treatment of anxiety symptoms [71]. The glucocorticoid decline associated with PTSD was previously thought to be a consequence rather than a cause of trauma, but in recent years has been shown to predict trauma, with studies showing that people who show reduced cortisol levels in the emergency room immediately after a traumatic event are those who ultimately develop PTSD [72]. A recent study using machine learning analyses showed that reduced cortisol levels collected in the emergency room after trauma accurately predicted a greater risk of later non-remitted PTSD [73]. Furthermore, in a large prospective cohort, low cortisol levels predicted subsequent fatigue, suggesting that it is a trait rather than a state marker [73]. Social phobia patients have significantly lower plasma cortisol levels compared with healthy controls [74]. Mice housed in an envy-like environment have increased anxiety-like behaviour, which is consistent with a decrease in serum corticosterone concentrations. Despite the complex relationship between cortisol or its equivalent, corticosterone, and the formation of fear- and anxiety-related symptoms in rodents, the neurobiological mechanisms are unclear [75]. Envy-like environments increase anxiety-like behaviours and decrease serum corticosterone concentrations.

In this study, mice raised in an envy-like environment demonstrated increased anxiety-like behaviour. In mice, an envy-like environment causes psychological stress, and thus, rearing in this environment may facilitate the creation of an animal model for anxiety disorders. The global lifetime prevalence of mood and anxiety disorders is estimated at 13 and 10%, respectively [76]. Currently, in the United States and Europe, anxiety disorders are the most common neuropsychiatric disorders [77,78], which constitute major health problems in the Western world in terms of medical costs, sick leave, disability, and premature death [79]. Nevertheless, an understanding of the aetiology of anxiety disorders remains incomplete because of several possible reasons, including the complexity and heterogeneity of anxiety disorders, which have multifactorial causation. Improving animal models is essential for furthering our understanding of the neurobiology of anxiety disorders and the development of effective treatments. As noted by many authors, the behavioural responses and brain mechanisms associated with anxiety states are crucial for survival and must therefore have evolved very early in mammalian development and are probably highly conserved [80].

Envy is an unpleasant emotion that is characterized by not only feelings of inferiority but also latent hostility and resentment towards others [62]. Envy usually arises from comparison with others who have what one desires, and its intensity is influenced by many factors [62]. The more similar someone is perceived to be to oneself, the greater the intensity of envy, and this increases when others’ advantages are related to areas of high self-relevance. Moreover, the intensity of envy increases with the perception that the benefit is unavailable [62]. Consistently high levels of envy are associated with higher levels of maladjustment and psychopathology [12]. Envy is positively correlated with depression, anxiety, somatization, obsessive–compulsive symptoms, and paranoia [12]. Thus, we showed that mouse models of anxiety disorders can be created by raising mice in the desired environments. Nevertheless, further research is required to establish our model as an actual mouse model for anxiety disorders.

Gamma-aminobutyric acid, the brain’s main inhibitory neurotransmitter, has long been considered to be the most important factor in the pathogenesis of anxiety [81]. The precise neurobiology underlying anxiety remains elusive and complex in nature. Roles of serotonin, noradrenaline, endorphins, and dopamine have been implicated in underlying anxiety symptoms [82].

The use of only male mice in our study may be considered a limitation. One of the most widely documented findings in psychiatric epidemiology is that women are significantly more likely than men to develop anxiety disorders in their lifetime [83,84]. Further studies are needed to examine whether similar results can be obtained using female mice housed in an envy-like environment. In this study, we performed envy-like rearing by presenting mice that were housed in cages with environmental enrichment. However, research is needed to clarify whether the same increase in anxiety-like behaviour and decrease in serum corticosterone concentration is observable in other envy-like rearing environments. As in previous mouse behavioural experiments, we used a sample size of 10, but individual differences may have led to bias. Further research is needed to increase the sample size and investigate the biological mechanisms of envy. In this study, mice were kept in an envy-like environment for 3 weeks to chronically stress them. A shorter period of exposure to the environment may have produced different results. Because the mice were naive, the control group in this study needed to be made aware of the enrichment devices. If they were not made aware of these, the control group might have exhibited different behaviour. In addition, it is necessary to establish experimental methods to examine whether chronic envy-like rearing induces mice to exhibit specific envy-like behaviours.

In conclusion, when mice are housed in an envy-like environment with an adjacent environmental enrichment cage, they exhibit increased anxiety-like behaviour and decreased serum corticosterone levels. Thus, mice recognize what is happening in neighbouring cages and experience psychological stress. This study provides scientific evidence that envy increases anxiety. With the breeding environment described in this experiment, an animal experimental model for anxiety disorders could be created.
